# Cost-effective analysis of hepatitis A vaccination in Kerala state, India

**DOI:** 10.1371/journal.pone.0306293

**Published:** 2024-06-27

**Authors:** Yogesh Krishnarao Gurav, Bhavani Shankara Bagepally, Natthakan Chitpim, Abhasnee Sobhonslidsuk, Mohan Digambar Gupte, Usa Chaikledkaew, Ammarin Thakkinstian, Montarat Thavorncharoensap

**Affiliations:** 1 Mahidol University Health Technology Assessment (MUHTA) Graduate Program, Mahidol University, Bangkok, Thailand; 2 Health Technology Assessment Group, ICMR-National Institute of Virology, Pune, Maharashtra, India; 3 Division of Non-Communicable Diseases, ICMR-National Institute of Epidemiology, Chennai, Tamil Nadu, India; 4 Social, Economic and Administrative Pharmacy Graduate Program, Faculty of Pharmacy, Mahidol University, Bangkok, Thailand; 5 Division of Gastroenterology and Hepatology, Department of Medicine, Faculty of Medicine Ramathibodi Hospital, Mahidol University, Bangkok, Thailand; 6 ICMR- National Institute of Epidemiology, Chennai, Tamil Nadu, India; 7 Social and Administrative Pharmacy Division, Department of Pharmacy, Faculty of Pharmacy, Mahidol University, Bangkok, Thailand; 8 Department of Clinical Epidemiology and Biostatistics, Faculty of Medicine Ramathibodi Hospital, Mahidol University, Bangkok, Thailand; Centers for Disease Control and Prevention, UNITED STATES

## Abstract

Several hepatitis A outbreaks have recently been reported in Kerala state, India. To inform coverage decision of hepatitis A vaccine in Kerala, this study aimed to examine the cost-effectiveness of 1) hepatitis A vaccination among children aged 1 year and individuals aged 15 years, and 2) serological screening of individuals aged 15 years and vaccination of susceptible as compared to no vaccination or vaccination without serological screening. Both live attenuated hepatitis A vaccine and inactivated hepatitis A vaccine were considered in the analysis. A combination of decision tree and Markov models with a cycle length of one year was employed to estimate costs and benefits of different vaccination strategies. Analysis were based on both societal and payer perspectives. The lifetime costs and outcomes were discounted by 3%. Our findings indicated that all strategies were cost-saving for both societal and payer perspectives. Moreover, budget impact analysis revealed that vaccination without screening among individuals aged 15 years could save the government’s budget by reducing treatment cost of hepatitis A. Our cost-effectiveness evidence supports the inclusion of hepatitis A vaccination into the vaccination program for children aged 1 year and individuals aged 15 years in Kerala state, India.

## Introduction

In 2016, the World Health Assembly (WHA) endorsed the Global Health Sector Strategy (GHSS) on viral hepatitis 2016–2021 and called for the elimination of viral hepatitis as a public health threat by 2030 [1]. Hepatitis A is a liver disease [2], caused by a hepatitis A virus (HAV) of the *picornaviridae* family [2]. The virus is transmitted through ingestion of contaminated food and water or even by direct contact with hepatitis A infectious person [3]. Symptom and severity of hepatitis A is strongly correlated with age [3]. While young children usually have asymptomatic infection, adolescents and adults may experience symptoms including fever, malaise, nausea, loss of appetite, abdominal discomfort, diarrhoea, dark-coloured urine, and jaundice [3]. Although the burden of hepatitis A is relatively low in high income countries [4], the incidence rate is still high in many middle-income (MICs) and low-income (LICs) countries [4]. In many MICs with a mixture of intermediate-and low-endemic levels or in transition from intermediate to low-endemic level [5] especially in India [6], increasing outbreak due to hepatitis A among adolescents and adults can be observed.

Vaccination with the two-dose inactivated hepatitis A vaccine or one dose of live attenuated hepatitis A vaccine is the effective method to prevent HAV infection [3]. According to the World Health Organization (WHO), “vaccination against hepatitis A virus be introduced into national immunization schedules for individuals aged ≥12 months, if indicated on the basis of: i) an increasing trend over time of acute hepatitis A disease, including severe disease, among older children, adolescents or adults; ii) changes in the endemicity from high to intermediate; and iii) considerations of cost-effectiveness”[6].

India has 29 states and 7 union territories [7], with a seroprevalence of 60%-80% of hepatitis A infection noted among children aged ≤ 5 years in various states in India [8, 9].

Among 29 Indian states, Kerala state reported the lowest HAV antibody seroprevalence rate (4.3%-10.3%) among children and individuals aged ≤ 15 years [8–10]. With the decreasing number of adolescents with prior exposure to HAV, several hepatitis A outbreaks occurred, specifically, with high attack rate among this population in Kerala [11]. Large out of pocket expenditure on treatment of hepatitis A disease was reported [12]. Public health research findings [9, 11, 13], and social media reports has consistently demanded the introduction of hepatitis A vaccination to the population in Kerala [14, 15]. Nevertheless, policy decision on such large-scale immunization program should be based on evidence-based information. Although previous published cost-effectiveness studies of hepatitis A vaccination suggested that hepatitis A vaccination is cost-effective in most developing countries [16–21], there is a lack of cost-effectiveness evidence of hepatitis A vaccination strategy to inform policy decision making in India. The present study was conducted to estimate the cost-effectiveness of various hepatitis A vaccination strategies compared to no vaccination among the population in Kerala state, India. The budget impact analysis for providing hepatitis A vaccine was also performed.

## Methods

### Interventions and comparator

In this study, the following three strategies of hepatitis A vaccination were evaluated among individuals aged 15 years: 1) no hepatitis A vaccination 2) hepatitis A vaccination, 3) screening for HAV antibody using anti-HAV IgG enzyme-linked immunosorbent assay (ELISA) screening test and then providing hepatitis A vaccination to susceptible individuals. First and second strategies were also evaluated in children aged 1 year. Both live attenuated hepatitis A vaccine and inactivated hepatitis A vaccine, which were available in the Indian market were considered in our study [22, 23].

### Model structure and assumptions

**Fig 1:**
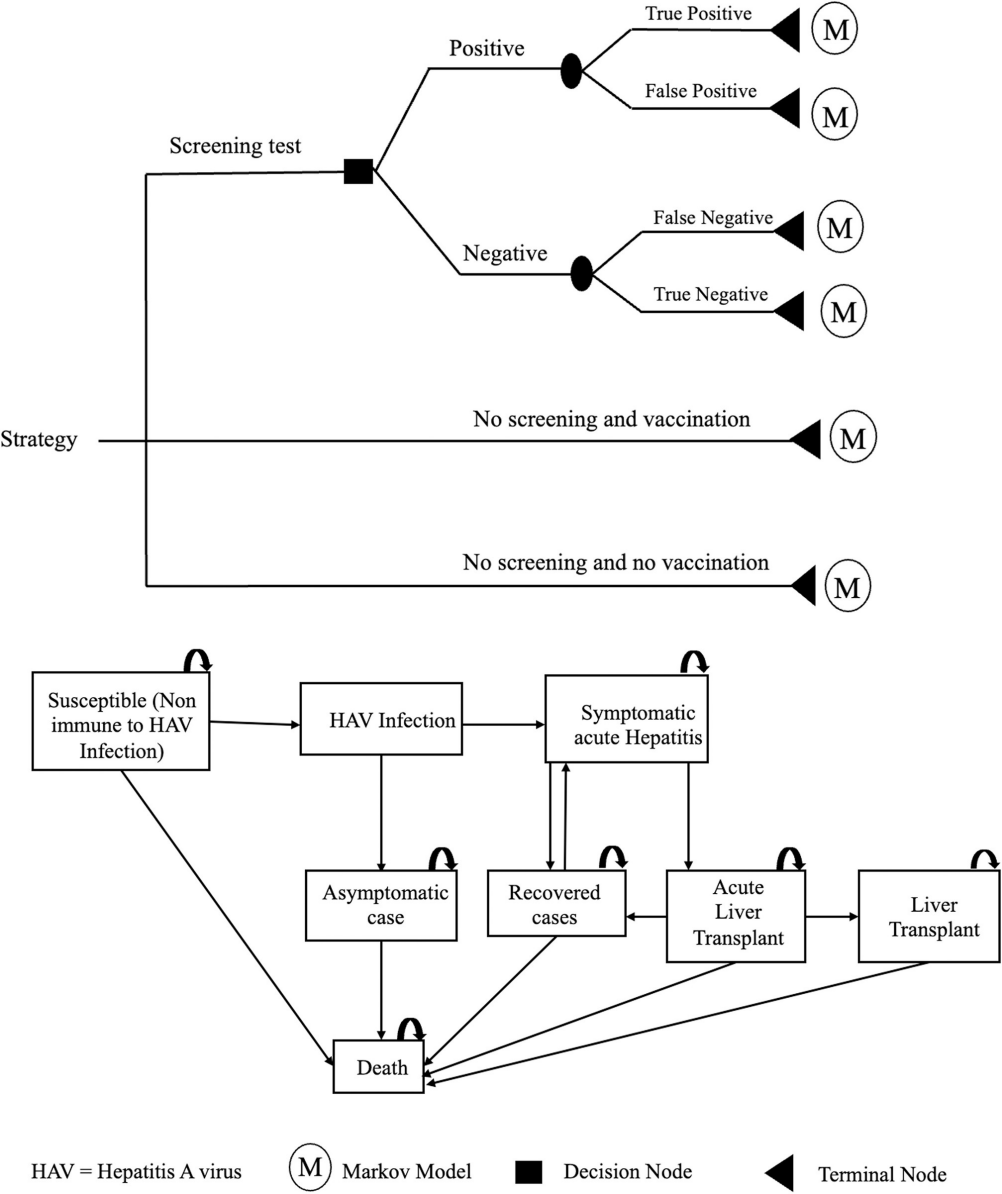
Model structure.

A combination of decision-tree and Markov models was employed to predict the lifetime costs and outcomes of each strategy based on societal and payer perspectives (Fig 1).

The model was developed based on the natural history of disease of HAV infection and was validated by clinical experts in India and Thailand. It consisted of eight mutually exclusive health states, including susceptible, hepatitis A infection, symptomatic acute hepatitis, asymptomatic case, recovered, acute liver failure, liver transplant, and death. A lifetime horizon was used with a cycle length of one year. A 3% discount rate was used for both costs and outcomes [24]. The model was developed and run in Microsoft Office Excel (Microsoft Corp., Redmond, WA).

The following assumptions were adopted in the analysis: 1) relapse would occur only once; 2) deaths from hepatitis A would only occur in hospitals due to acute liver failure/fulminant hepatitis; 3) cost of adverse effects due to vaccination was not considered as they were very rare and mild; 4) study population would get the vaccination and screening test at primary (35%), secondary (35%) and tertiary health facilities (30%); and 5) one parent would accompany children aged 1 year and individuals aged 15 years during vaccination and screening.

### Model input parameters

The model input parameters are described and summarised in Table 1.

**Table 1:** Model parameters.

Parameter	Mean	SE	Distribution	Source
**Transitional probabilities (Epidemiological and clinical)**				
Susceptible individuals to hepatitis A infection:				
1–10 years	0.0979	0.0207	Beta	[10]
11–15 years	0.1324	0.0441	Beta	[10]
≥ 16 years	0.3100	0.0486	Beta	[10]
Hepatitis A infection to symptomatic acute hepatitis:				
≤ 4 years	0.0695	0.0257	Beta	[25]
5–9 years	0.3100	0.0481	Beta	[25]
10–17 years	0.5069	0.0453	Beta	[25]
≥18 years	0.5734	0.0353	Beta	[25]
Hepatitis A infection to asymptomatic:				
≤ 4 years	0.9305	0.0257	Beta	[25]
5–9 years	0.6900	0.0481	Beta	[25]
10–17 years	0.4931	0.0453	Beta	[25]
≥18 years	0.4266	0.0353	Beta	[25]
Symptomatic acute hepatitis to spontaneous recovery	0.9900	0.0099	Beta	[26]
Symptomatic acute hepatitis to acute liver failure	0.0100	0.0099	Beta	[26]
Spontaneous recovered cases to symptomatic hepatitis cases (relapse)	0.1393	0.0355	Beta	[27]
Acute liver failure to liver transplant	0.3500	0.0475	Beta	[26]
Acute liver failure to spontaneous recovery	0.5500	0.0495	Beta	[26]
Acute liver failure to death	0.1000	0.0299	Beta	[26]
Death among liver transplant cases with in 1 year	0.1800	0.0382	Beta	[28]
Death among liver transplant cases from 2–3 year after transplant	0.0717	0.0398	Beta	[28]
Death among liver transplants from 4 year onwards for each subsequent year	0.0209	0.0299	Beta	[28]
Death among general population by age	0.0090	0.0009	Beta	[7]
**Effectiveness parameters**				
Screening test (HAV ARCHITECT IgG assay)				
Sensitivity of screening test	0.9980	0.0032	Beta	[29]
Specificity of screening test	0.9917	0.0032	Beta	[29]
Effectiveness of hepatitis A vaccines				
Relative risk of attenuated vaccine	0.0700	0.0360	Beta	[30]
Relative risk of inactivated vaccine	0.0900	0.0690	Beta	[30]
Program coverage				
Hepatitis A vaccination	0.9	-	-	[31]
Screening test	0.9	-	-	[31]
**Cost parameters (value in 2023 Indian Rupees ₹)**				
Cost of screening test (HAV ARCHITECT IgG assay)	600.00	600.00	Gamma	[32]
Cost of Attenuated hepatitis A vaccine (one dose)	1010.00	1010.00	Gamma	[22, 23]
Cost of Inactivated hepatitis A vaccine (one dose)	1,263.00.00	1263.00	Gamma	[22, 23]
Vaccine delivery cost	502.77	502.77	Gamma	[33]
Travel cost to primary health center facility	20.29	20.29	Gamma	[34]
Travel cost to rural hospital/district hospital facility	115.94	115.94	Gamma	[34]
Travel cost to tertiary care hospital facility	423.19	423.19	Gamma	[34]
Direct medical cost for symptomatic acute hepatitis	12,749.87	12749.87	Gamma	[12]
Direct medical cost for acute liver failure	190,565.59	190,565.59	Gamma	[35]
Direct medical cost for liver transplant cost	3,976,130.64	3,976,130.64	Gamma	[28]
Direct medical cost for liver transplant maintenance cost for 1st year	360,635.78	360,635.78	Gamma	[36]
Direct medical cost for liver transplant maintenance cost for subsequent year	184, 394.64	184, 394.64	Gamma	[36]
**Utility**				
Symptomatic acute hepatitis	0.43	0.03	Beta	[18]
Acute liver failure	0.43	0.03	Beta	[18]
Liver transplant (1^st^ year)	0.57	0.10	Beta	[37, 38]
Liver transplant (2^nd^ year onward)	0.67	0.10	Beta	[37, 38]
Utility score for Indian population age <20	0.93	0.02	Beta	[39]
Utility score for Indian population age 20–29 years	0.91	0.02	Beta	[39]
Utility score for Indian population age 30–39 years	0.87	0.03	Beta	[39]
Utility score for Indian population age 40–49 years	0.83	0.04	Beta	[39]
Utility score for Indian population age 50–59	0.79	0.04	Beta	[39]
Utility score for Indian population age 60–69	0.72	0.06	Beta	[39]
Utility score for Indian population age >70	0.57	0.14	Beta	[39]

### Cost

All costs were derived from Indian published studies [11, 12, 28, 33, 35, 36], specifically in Kerala State [12, 36] (Table 1). Direct medical costs (i.e., costs of hospitalization, consultation, drugs, and investigation), direct non-medical costs (i.e., costs of food, transportation, and caregiver) and indirect costs (i.e., income loss by the parents or caretakers) for HAV infection were derived from the study conducted in Kerala [12]. It was assumed that travel costs and income loss were calculated for one parent/caretaker. The vaccine delivery cost was derived from a study conducted in India [32]. Vaccine cost was derived for December 2023 from the vaccine distributor agency in India through India Mart [22]. The screening test cost was considered from a private laboratory [32], including human resource and laboratory costs. It should be noted that the cost of Hepatitis A vaccination implementation was not included in our study based on the assumption that the delivery of Hepatitis A vaccination will be integrated with routine EPI immunization at health facilities. Therefore, the costs of program implementation were considered minimal, as compared to the costs of vaccine and cost of vaccine delivery.

All costs were converted to year 2023 by using the consumer price index specific for Kerala state [40]

### Effectiveness of interventions

As shown in Table 1, the efficacy of inactivated hepatitis A vaccine and live-attenuated hepatitis A vaccine was derived from a systematic review [30]. The vaccination coverage rate was considered 90% based on the WHO-SEARO fact sheet 2023, which informs the coverage for DTP-Hib-HepB vaccination coverage in Kerala as ≥ 90% [31].

### Utility

In our study, utilities of susceptible individual and asymptomatic infection were based on the population norms in India by age. Due to limited data in India, utility values related to liver transplants, symptomatic hepatitis, and acute liver failure were derived from the published studies in other countries. It should be noted that both acute hepatitis and acute liver failure are severe medical conditions [41]. However, the distinction between acute hepatitis (AH) and acute liver failure (ALF) can be challenging due to their overlapping clinical presentations [42]. Given the limited data, we used the same utility values for both health states in our analysis due to the similar symptoms. In addition, it should be noted that the utility values for liver transplants, symptomatic hepatitis, and acute liver failure presented in Table 1 were adjusted by population norms in India to reflect the utility of each health stage by age.

### Result presentation

Total costs, life years (LYs), and QALYs for each screening strategy were estimated over lifetime period. The results were presented as the incremental cost-effectiveness ratio (ICER) in terms of cost per quality-adjusted life year (QALY) gained. A cost-effectiveness threshold of one gross domestic product (GDP) per capita or approximately ₹ 111,927 per QALY [43] was applied.

### Uncertainty analysis

One-way and probabilistic sensitivity analyses (PSA) were performed. One-way sensitivity analysis was conducted to investigate the effects of different input parameters. Wherever available, standard errors (SEs) were used in sensitivity analysis. However, for parameters lacking SE data, we performed the sensitivity analysis by varying each parameter by ±25%. The results for one-way sensitivity analysis were presented as Tornado diagrams. PSA was performed by running 1,000 Monte Carlo simulations. The results of the PSA were presented as the cost-effectiveness acceptability curve (CEAC) by using one-time GDP per capita as a threshold.

### Budget impact analysis

Budget impact analysis (BIA) was conducted as per the recommendations by International Society for Pharmacoeconomics and Outcomes Research (ISPOR) task force report [44]. The analysis was performed from a payer perspective for a time horizon of five years from 2023–2027, considering the financial year in India, which counted from the month of April to March. The total number of children aged 1 year and individuals aged 15 years living in Kerala state, India, in the year 2023 was 460,000 and 502,600, respectively [45]. Annual cases of hepatitis and its complications were estimated from the model. Total annual vaccine costs were calculated as the products of cost per vaccine dose, predicted vaccination coverage, number of target population, number of doses, and predicted wastage factor [46, 47]. As per the WHO, the wastage factor for vaccination was considered as 5% [47]. For calculating the budget impact, the difference in total direct medical cost of hepatitis A treatment between vaccination and no vaccination were considered. Analysis was done considering the immunization coverage of 90% [31].

## Results

### Interventions among individual aged 15 years

Compared to no vaccination, hepatitis A vaccination, either with screening or without screening, resulted in cost-saving (i.e., reduced cost and increased QALY gained). As shown in Table 2, screening together with inactivated hepatitis A vaccination resulted in cost-saving with the ICERs of ₹ -23,599 per QALY gained for a societal perspective and ₹-17,108 per QALY gained for a payer perspective. Similarly, vaccination with inactivated hepatitis A vaccination without screening was found to be cost-saving for both societal (₹-87,382 per QALY gained) and payer perspective (₹-61,989 per QALY gained) (Table 2). Similar results were also found for live attenuated vaccine (S1 Table in S1 Text).

**Table 2:** Cost-utility analysis results classified by hepatitis A inactivated vaccine strategies and perspective among individuals aged 15 years.

Results	Societal perspective			Payer perspective results		
	No vaccination	Inactivated hepatitis A vaccination without screening	Screening and inactivated hepatitis A vaccination	No vaccination	Inactivated hepatitis A vaccination without screening	Screening and inactivated hepatitis A vaccination
Cost (₹)	45,046.17	28,060.90	27,926.80	32,738.47	18,598.41	20,327.69
LY^#^	24.17	24.57	24.35	24.17	24.57	24.35
QALY^#^	18.64	18.84	19.37	18.64	18.84	19.37
Incremental cost (₹)	NA	-16,985.28	-17,119.37	NA	-12,049.29	-12,410.78
Incremental LY^#^	NA	0.40	0.18	NA	0.40	0.18
Incremental QALY^#^	NA	0.19	0.73	NA	0.19	0.73
ICER^#^	NA	-45,512	-97,665	NA	-30,158	-70,803
(₹ per LY^#^ gained)						
ICER^#^	NA	-87,382	-23,599	NA	-61,989	-17,108
(₹ per QALY^#^ gained)						

^#^Abbreviations: Life years (LY); Quality adjusted life years (QALY); Incremental cost-effectiveness ratio (ICER), Not applicable (NA)

### Interventions among children aged 1 year

Hepatitis A vaccination among children aged 1 year as compared to no vaccination resulted in cost-saving (i.e., decreased cost and increased QALY gained) (Table 3 and S2 Table in S1 Text). For inactivated hepatitis A vaccination, the ICERs ranged from ₹-78,229 per QALY gained for a societal perspective and ₹-53,282 per QALY gained for a payer perspective (Table 3).

**Table 3:** Cost-utility analysis results classified by hepatitis A inactivated vaccine strategies and perspective among children aged 1 year.

Results	Societal perspective		Payer perspective	
	No vaccination	Inactivated Vaccine	No vaccination	Inactivated Vaccine
Cost (₹)	30,328.23	19,116.30	21,005.12	14,413.66
LY^#^	27.16	27.18	27.16	27.18
QALY^#^	21.68	21.82	21.68	21.82
Incremental cost (₹)	NA	-11,211.93	NA	-7,636.49
Incremental LY^#^	NA	0.02	NA	0.02
Incremental QALY^#^	NA	0.14	NA	0.14
ICER^#^ (₹ per LY^#^ gained)	NA	-634,633	NA	-432,252
ICER^#^ (₹ per QALY^#^ gained)	NA	-78,229	NA	-53,282

^#^Abbreviations: Life years (LY); Quality adjusted life years (QALY); Incremental cost-effectiveness ratio (ICER), Not applicable (NA)

### One-way sensitivity analysis

Results of the one-way sensitivity analysis are displayed in S1-S3 Figs in S1 Text. Among individuals aged 15 years receiving the live attenuated vaccine (S1 Fig in S1 Text), the most sensitive input parameters for a societal perspective were the probability of the symptomatic acute hepatitis to acute liver failure, discounting of outcome and probability of hepatitis A infection to recovery health stage while those of the payer perspective were probability of the symptomatic acute hepatitis to acute liver failure, probability of acute liver failure to liver transplant. For individuals aged 15 years receiving the inactivated hepatitis A vaccine, the probability of the symptomatic acute hepatitis to acute liver failure and acute liver failure getting liver transplant were the most sensitive input parameters from both societal and payers perspectives (S2 Fig in S1 Text). However, for screening and vaccination with live attenuated vaccine and screening and vaccination with inactivated vaccine, the most sensitive input parameters from both societal and payer perspectives were the probability of negative predictive value of screening test and the probability of screening test being positive for 1–15 years.

Among children aged 1 year receiving either the live attenuated vaccine or inactivated vaccine, the most sensitive input parameters both by societal and payers perspectives were the probability of symptomatic acute hepatitis A to acute liver failure and probability of acute liver failure getting a liver transplant (S3 Fig in S1 Text). However, for children receiving inactivated hepatitis A vaccination, the most sensitive parameters both by societal and payers perspective were the probability of symptomatic acute hepatitis A to acute liver failure and probability of acute hepatitis infection to asymptomatic among ≤ 4 years.

### Probabilistic sensitivity analysis results

**Fig 2:**
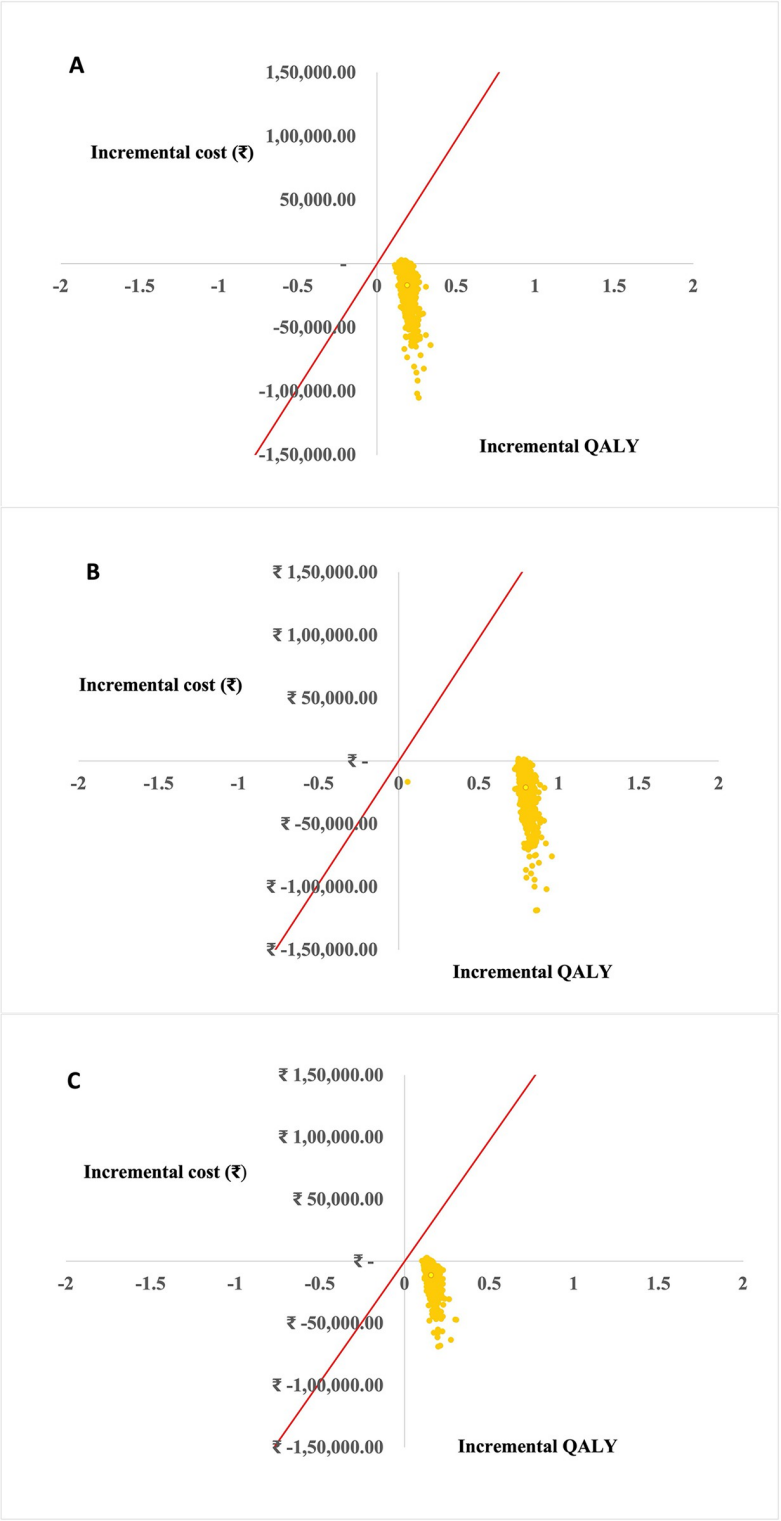
Cost-effectiveness plane by a societal perspective A) among 15 years receiving inactivated hepatitis A vaccination without screening strategy B) among 15 years with screening and inactivated hepatitis A vaccination C) Child aged 1 year receiving inactivated hepatitis A vaccination.

**Fig 3:**
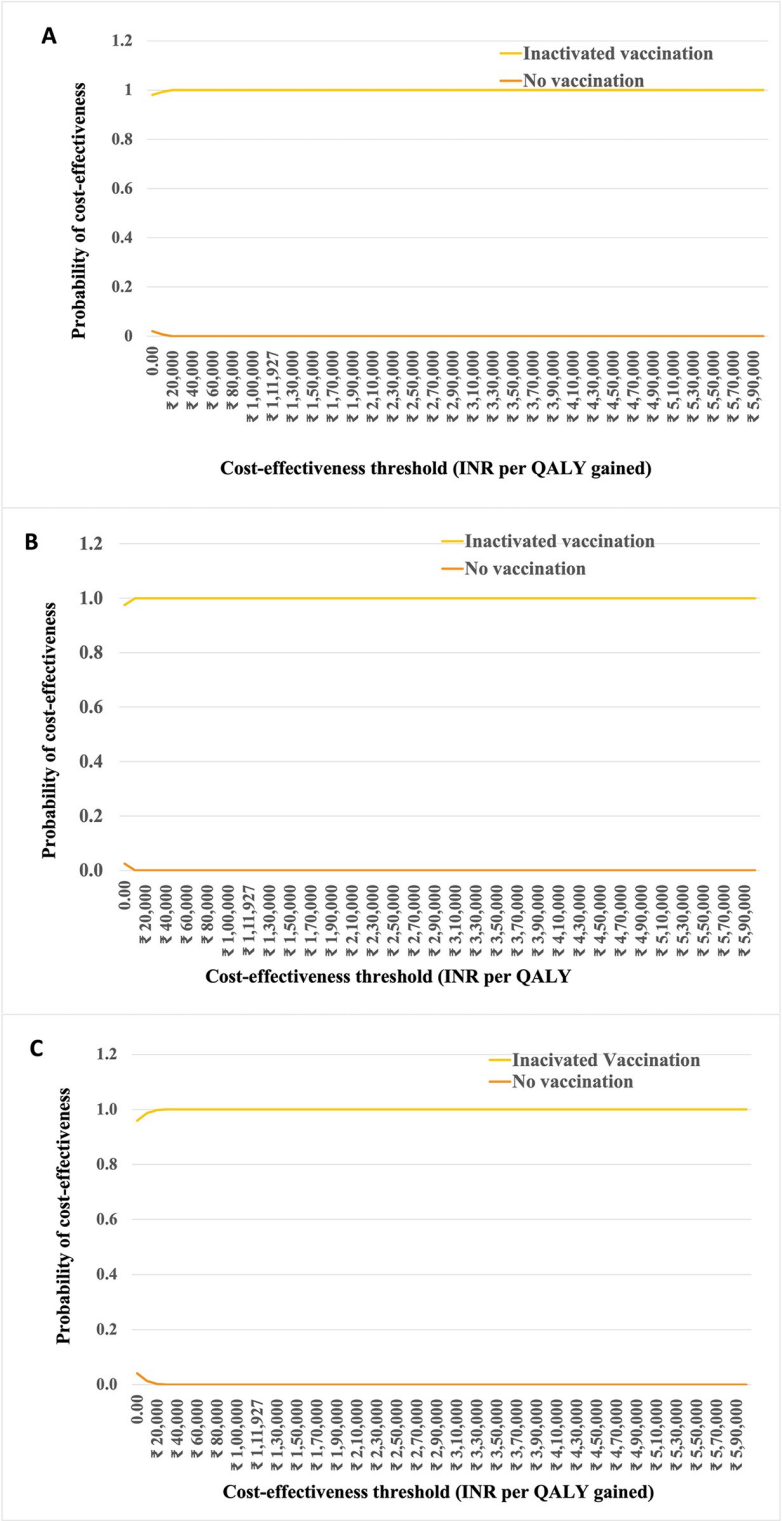
Cost-effectiveness acceptability curve by a societal perspective. A) among 15 years receiving inactivated hepatitis A vaccination without screening strategy B) among 15 years with screening and inactivated hepatitis A vaccination C) Child aged 1 year receiving inactivated hepatitis A vaccination.

Based on PSA results, the cost-effectiveness plane (Fig 2) demonstrated that the majority of simulations were in the south-east quadrant, suggesting that all inactivated hepatitis A vaccination options would save more money, while offering slightly more health outcome compared to no vaccination under societal perspective. Moreover, the cost-effectiveness acceptability curve showed that all inactivated hepatitis A vaccination options had the highest probability of being cost-effective at threshold of one GDP per capita (₹ 111,927) are displayed in Fig 3.

Probabilistic sensitivity analysis results for live attenuated vaccines (both societal and payer perspective) and inactivated vaccine (payer perspective) are shown in S3 and S4 Tables in S1 Text, respectively. These results are depicted in the form of cost-effectiveness planes as shown in S4-S6 Figs in S1 Text, respectively.

Based on both societal (Fig 3) and payer perspectives, among individuals aged 15 years, vaccination with live attenuated vaccine and inactivated vaccination had probability being cost-effective is about 100% (S7-S9 Fig in S1 Text). Screening and live attenuated vaccination strategy among individual aged 15 years had 99%-100% chance being cost-effective, while screening and inactivated vaccination strategy had probability being cost-effective about 99%-100% at the cost-effectiveness threshold in India. Among children aged 1 year, vaccination with live attenuated vaccine and inactivated vaccine was found to be 100% cost-effective (S7-S9 Figs in S1 Text).

### Budget impact analysis of vaccination strategies

Among individual aged 15 years, assuming 90% vaccination coverage and screening, a total incremental budget for vaccination with inactivated vaccine, and live attenuated vaccine were estimated to be approximately ₹ -5,872 million and ₹ -10,553 million, respectively (Table 4). However, the incremental budget would be ₹ -5,622 million and ₹ -9,591 million for screening and vaccination with inactivated or live attenuated vaccination, respectively. Among children aged 1 year, a total incremental budget for implementing the inactivated vaccine, and live attenuated vaccine were approximately ₹ 6,328 million and ₹ 1,992 million, respectively (Table 4).

**Table 4:** Budget impact analysis for hepatitis A vaccination strategy among individuals aged 15 years and children aged 1 year (million ₹).

**Among individual aged 15 years**														
**Fiscal year**	**Hepatitis A vaccination strategy**								**Screening and hepatitis A vaccination**					
	**Total budget**					**Incremental budget**			**Total budget**			**Incremental budget**		
	**No vaccination**		**Inactivated**	**Live attenuated**		**Inactivated**		**Live attenuated**	**Inactivated**		**Live attenuated**	**Inactivated**		**Live attenuated**
1	812		1,570	696		758		-116	1,750		991	938		179
2	1,490		1,650	759		160		-731	1,820		1,050	330		-440
3	3,080		1,830	904		-1,250		-2,176	1,900		1,110	-1,180		-1,970
4	4,320		2,030	1,060		-2,290		-3,260	2,000		1,190	-2,320		-3,130
5	5,500		2,250	1,230		-3,250		-4,270	2,110		1,270	-3,390		-4,230
Total	15,202		9,330	4,649		-5,872		-10,553	9,580		5,611	-5,622		-9,591
**Among children aged 1 year**														
**Fiscal year**		**Total budget**								**Incremental budget**				
		**No vaccination**			**Inactivated**		**Live attenuated**			**Inactivated**			**Live attenuated**	
1		33			1,520		652			1,487			619	
2		67			1,520		654			1,453			587	
3		142			1,530		660			1,388			518	
4		429			1,560		689			1,131			260	
5		731			1,600		739			869			8	
Total		1,402			7,730		3,394			6,328			1,992	

## Discussion

As per our knowledge, this is the first study examining the cost-effectiveness of hepatitis A vaccination in Kerala state, and also in India. Among individual aged 15 years, vaccination program with live attenuated hepatitis A vaccination or inactivated hepatitis A vaccination resulted in lower cost and higher QALY gained, suggesting that vaccination either with inactivated or live-attenuated vaccine would be a cost-saving intervention. In addition, screening and providing live attenuated hepatitis A or inactivated hepatitis A vaccination strategy for susceptible individuals was found to be a cost-saving intervention for individuals aged 15 years. Similarly, vaccination with either live attenuated hepatitis A vaccination or inactivated hepatitis A vaccination seemed to be cost-saving among children aged 1 year for both societal and payer perspectives.

It was highlighted that our results were similar to those of studies conducted in middle-income countries including countries in Asia region, which indicating that hepatitis A vaccination among children were likely to be cost-effective or even cost-saving [18, 20, 21, 48]. The reason that our results was in line with the studies conducted in middle income countries including China and Indonesia is probably due to similar context related to endemicity of hepatitis A. With improvement in living and economic condition over the past decades, HAV infection is shifting from early childhood to adolescence and young adulthood reflecting epidemiological transition of hepatitis A in Kerala state, India [9, 49] resulting in an increased risk of getting hepatitis A infection [11, 50]. Previous studies [48, 51] as well as a recent systematic review [21] on economic evaluation of hepatitis A vaccination suggested that incidence of hepatitis A is one of the most influential factors in the cost-effectiveness of hepatitis A vaccination. In addition, our results were consistent with the WHO recommendations that vaccination against hepatitis A be integrated into the national immunization schedule for children aged ≥ 1 year on the basis of incidence of hepatitis A, change in endemicity from high to intermediate, and considering the cost-effectiveness of vaccination strategy [5].

On the other hand, our findings are inconsistent with the earlier study conducted in Belgium which reported that the screening and vaccination compared with no vaccination would not be cost-effective for adult population [52]. This inconsistent result could be explained by the difference in the epidemiological circumstance of the two countries, where vaccination was not be an cost-effective option for low endemic area.

Although our results indicated that hepatitis A vaccination among individuals aged 15 years with or without screening would be a cost-saving, however, screening would require professionally trained human resources and skills. In addition, effective plan is essential to the implementation of mass screening and vaccination. Thus, vaccination without screening seemed to be an attractive choice, as it would be more feasible for implementation.

Nevertheless, the budget impact for implementing vaccination program among children aged 1 year with live attenuated and inactivated vaccine were estimated at ₹ 1,992 million and ₹ 6,328 million, respectively. As of the fiscal year 2023, the Government of Kerala has allotted a budget of ₹ 99,350 million to Health and Family Welfare [53]. Based on our analysis, the Government of Kerala would need to allot 0.68% extra budget for introduction of live attenuated vaccine to child aged 1 year as compared to 1.56% extra budget for introduction of inactivated hepatitis A vaccine every year for five years (year 2023 to year 2027), given that the Government budget in the next 5 years was fixed.

However, introduction of either live attenuated vaccine or inactivated vaccine for individual aged 15 years would save 2.12% and 1.18% of the Government budget (for fiscal year 2023 to 2027), each year respectively by a payer perspective. Similarly, screening with live attenuated vaccination or screening with inactivated hepatitis A vaccination among individual aged 15 years would save approximately 1.93% and 1.13% of the Government budget every year, respectively by a payer- perspective as it could also save budget spent on treatment of hepatitis in the future. It should be noted that this budget impact analysis assumed 90% vaccination coverage (considering immunization coverage in Kerala state close to 90%) However, in reality, the coverage and compliance may differ from region to region in Kerala, India.

Based on the cost-effectiveness and budget impact analysis results, the introduction of both inactivated vaccine and live attenuated vaccine among child aged 1 year and individual aged 15 years would be an attractive choice due to cost-saving options. However, the budget for implementing vaccination program in child aged 1 year with inactivated vaccine was higher than those with live attenuated vaccine. Although the use of live attenuated vaccine resulted in less budget impact, as compared to inactivated vaccine, the careful consideration should be given on the safety assessment of the vaccine and its global utilization. As recommended by the WHO, more studies on the use of live attenuated vaccines [5] in the community were warranted while the use of inactivated hepatitis A vaccine is the most widely acceptable vaccine globally [5].

In addition, our study found that probability of the symptomatic acute hepatitis to acute liver failure, discounting of outcome, discounting of cost and probability of acute liver failure to liver transplant were the most sensitive parameters while earlier studies reported that the vaccine price, mortality rate and discount rate were the sensitive parameters [18, 20, 48]. It is well noted that the exact choice of discount rate is one of the most influential parameters for economic evaluation of vaccine as cost is more concentrated in early time period and health benefits are seen over longer period [52]. Nevertheless, it should be noted that many of the important parameters involved in our analysis were derived from Indian’s studies and statistics. Specifically, several epidemiological parameters like the seroprevalence of hepatitis A, death rate and the cost parameters (direct medical cost, direct nonmedical cost and indirect cost) were derived from the studies published in the Kerala state. However, it should be noted that some local information such as transitional probability of hepatitis A infection, utilities associated with hepatitis A health state were still lacking and were warranted as to generate the most reliable costs effectiveness results.

It was noteworthy to address the limitations in our study. Firstly, the findings of this study may not be generalized to whole India considering the heterogeneity of study population. However, we can apply state-specific parameters to examine cost-effectiveness of hepatitis A vaccine in other states in the future studies. Secondly, we assumed that all the persons would get the vaccine at fixed time points (2 doses as per the schedule) with the coverage of 90%. Future study should be further investigated on different scenarios with different doses and adherence rates. It could be seen from a study in Argentina which performed various possible scenarios related to receiving the second dose of vaccination at 18 month or 72 months. The study revealed that with one dose of vaccination, if duration of protection is less than anticipated giving second dose of hepatitis A vaccine would be more cost effective [54]. In addition, cost of hepatitis A vaccination implementation was not included in our study. Given that the cost of vaccination implementation is minimal as it will be added on to the existing EPI program and that the findings indicated that all vaccination strategies are cost-saving, we believed that if vaccination implementation cost were to considered the conclusions of cost-effective findings should remain unchanged.

At present, the National Technical Advisory Group on Immunization (NTAGI) by the Government of India recommended the use of hepatitis A vaccination only in the context of epidemic control and for individual use and indicated need of doing further cost effectiveness studies on hepatitis A vaccination [55]. However, Indian Academy of Paediatrics (IAP) has advocated inclusion of hepatitis A vaccination in immunization schedule [13].

## Conclusion

Hepatitis A vaccination was cost-saving in both children aged 1 year and individuals aged 15 years with or without screening. Our findings provide supporting evidences for the inclusion of hepatitis A vaccination into the vaccination program in Kerala state, India.

## Supporting information

S1 Text Supplemental data file. (DOCX)

S1 Data Data used to generate graphs. (XLSX)

## References

[1] World Health Organization. Global hepatitis report. Available from: http://www.who.int/hepatitis/publications/global-hepatitis-report2017/en/.

[2] Hollinger FB, Ticehurst JR. Hepatitis A Virus. In: Fields BN, Knipe DM, Howley PM, editors. Fields virology. 3 ed, Philadelphia: Wolters Kluwer Health; 2013. p. 735–82.

[3] World Health Organization. Hepatitis A Geneva: World Health Organization; 2018 [cited 2018 22 October]. Available from: http://who.int/mediacentre/factsheets/fs328/en/.

[4] Cao G, Jing W, Liu J, Liu M. The global trends and regional differences in incidence and mortality of hepatitis A from 1990 to 2019 and implications for its prevention. Hepatology international. 2021;15(5):1068–82. doi: 10.1007/s12072-021-10232-4 34345993 PMC8514357

[5] World Health Organization. WHO position paper on hepatitis a vaccines–October 2022 Weekly Epidemiological Record. 2022;97:493–512. Available from: https://www.who.int/publications/i/item/who-wer9740-493-512

[6] Agrawal A, Singh S, Kolhapure S, Hoet B, Arankalle V, Mitra M. Increasing burden of hepatitis A in adolescents and adults and the need for long-term protection: a review from the Indian subcontinent. Infectious Diseases and Therapy. 2019;8:483–97. doi: 10.1007/s40121-019-00270-9 31679118 PMC6856242

[7] India Census. Sample Registration System (SRS) Statistical Report 2020 Office of the Registrar General and Census Commissioner, India. Available from: https://censusindia.gov.in/census.website/node/378

[8] Mittal S, Rastogi A, Rastogi A, Kumar N, Talukdar B, Kar P. Seroprevalence of hepatitis A in children—implications for hepatitis A vaccine. Tropical gastroenterology. 1998;19(3):120–121. 9828714

[9] Mathur P, Arora N. Epidemiological transition of hepatitis A in India: issues for vaccination in developing countries. Indian Journal of Medical Research. 2008;128(6):699–704. 19246792

[10] Mall M, Rai R, Philip M, Naik G, Parekh P, Bhawnani S, et al. Seroepidemiology of hepatitis A infection in India: changing pattern. Indian journal of gastroenterology: official journal of the Indian Society of Gastroenterology. 2001;20(4):132–135. 11497169

[11] Rakesh PS, M K S. 84 outbreaks of Hepatitis A in last five years in Kerala State–are we resigning to fate? National Journal of Research in Community Medicine. 2017;6(3):267–270. Avaialble from: https://pubmed.ncbi.nlm.nih.gov/29355152

[12] Rakesh PS, Retheesh R, Chandran R, Sadiq A, Ranjitha S. Out-of-pocket expenditure due to hepatitis A disease: A study from Kollam district, Kerala, India. The Indian Journal of Medical Research. 2017;146(3):426–429. doi: 10.4103/ijmr.IJMR_275_16 ; PubMed Central PMCID: PMC5793480. http://www.ncbi.nlm.nih.gov/pmc/articles/pmc5793480/29355152 PMC5793480

[13] Vashishtha VM, Kalra A, Bose A, Choudhury P, Yewale VN, Bansal CP, et al. Indian Academy of Pediatrics (IAP) recommended immunization schedule for children aged 0 through 18 years, India, 2013 and updates on immunization. Indian pediatrics. 2013;50(12):1095–108. Epub 2014/01/15. doi: 10.1007/s13312-013-0292-9 . https://acvip.org/professional/columns/pdf/2013.pdf 24413503

[14] Pereira I. Time for hepatitis A vaccine: study [Internet]. 2016; 23 September 2016. Available from: https://www.thehindu.com/news/national/kerala/Time-for-hepatitis-A-vaccine-study/article14026568.ece

[15] Rakesh PS, Nayar KR, Shaffib M, Graceb C. Do we need to consider Universalising Hepatitis A Vaccine in Kerala, India? J of Health Systems. 2016;2(1):1 https://www.researchgate.net/publication/313106088_Do_we_need_to_consider_Universalising_Hepatitis_A_Vaccine_in_Kerala_India

[16] Lopez E, Debbag R, Coudeville L, Baron-Papillon F, Armoni J. The cost-effectiveness of universal vaccination of children against hepatitis A in Argentina: results of a dynamic health-economic analysis. Journal of gastroenterology. 2007;42(2):152–160. Epub 2007/03/14. doi: 10.1007/s00535-006-1984-x . 10.1007/s00535-006-1984-x 17351805

[17] Quezada A, Baron-Papillon F, Coudeville L, Maggi L. Universal vaccination of children against hepatitis A in Chile: a cost-effectiveness study. Revista panamericana de salud publica. 2008;23(5):303–312. Epub 2008/05/31. doi: 10.1590/s1020-49892008000500002 . 10.1590/s1020-49892008000500002 18510790

[18] Zhuang GH, Pan XJ, Wang XL. A cost-effectiveness analysis of universal childhood hepatitis A vaccination in China. Vaccine. 2008;26(35):4608–4616. Epub 2008/07/04. doi: 10.1016/j.vaccine.2008.05.086 . 10.1016/j.vaccine.2008.05.086 18597903

[19] Pan XJ, Feng YM, Zhuang GH. Cost-utility analysis on universal childhood hepatitis A vaccination in regions with different anti-HAV prevalence rates of China. Zhonghua liu xing bing xue za zhi. 2012;33(8):862–866. Epub 2012/09/13. . 22967346

[20] Suwantika AA, Beutels P, Postma MJ. Cost-effectiveness of hepatitis A vaccination in Indonesia. Hum Vaccin Immunother. 2014;10(8):2342–2349. Epub 2014/11/27. doi: 10.4161/hv.29353 ; PubMed Central PMCID: PMC4896775. Avaialble from: https://doi.org/10.4161%2Fhv.29353 25424941 PMC4896775

[21] Gurav YK, Bagepally BS, Thakkinstian A, Thavorncharoensap M. Economic evaluation of hepatitis A vaccines by income level of the country: A systematic review. Indian Journal of Medical Research. 2022;156(3):388–410. doi: 10.4103/ijmr.IJMR_1631_20 36629171 PMC10101351

[22] IndiaMart. Immunization and vaccination Drug. Hepatitis A vaccine. 2023. Available from: https://www.indiamart.com/proddetail/freeze-dried-live-attenuated-hepatitis-a-vaccine-23470263388.html

[23] Modern drug house: Trader exporter and supplier of pharmaceutical vaccine and injectable vaccine. 2022. Available from: https://www.moderndrughouse.in/profile.html

[24] Department of Health Research. Health Technology Assessment Manual by Health Technology Assessment India (HTAIn), Dept of Health Research, Govt of India. 2018. https://www.researchgate.net/publication/366066447_Health_Technology_Assessment_in_India_A_Manual

[25] Armstrong GL, Bell BP. Hepatitis A virus infections in the United States: model-based estimates and implications for childhood immunization. Pediatrics. 2002;109(5):839–845. doi: 10.1542/peds.109.5.839 11986444

[26] Shin EC, Jeong SH. Natural History, Clinical Manifestations, and Pathogenesis of Hepatitis A. Cold Spring Harbor perspectives in medicine. 2018;8(9):A031708. Epub 2018/02/15. doi: 10.1101/cshperspect.a031708 . https://doi.org/10.1101%2Fcshperspect.a031708 29440324 PMC6120688

[27] Glikson M, Galun E, Oren R, Tur-Kaspa R, Shouval D. Relapsing hepatitis A. Review of 14 cases and literature survey. Medicine. 1992;71(1):14–23. doi: 10.1097/00005792-199201000-00002 . 10.1097/00005792-199201000-00002 1312659

[28] Nagral S, Nanavati A, Nagral A. Liver transplantation in India: at the crossroads. J Clin Exp Hepatol. 2015;5(4):329–40. doi: 10.1016/j.jceh.2015.11.001 . 10.1016/j.jceh.2015.11.001 26900275 PMC4723645

[29] Abbot diagnostic division. Architect system HAVAB-IgG. Available from: https://www.ilexmedical.com/files/PDF/HAVAb-IgG_AEC.pdf

[30] Irving GJ, Holden J, Yang R, Pope D. Hepatitis A immunisation in persons not previously exposed to hepatitis A. The Cochrane database of systematic reviews. 2012;(7):Cd009051. Epub 2012/07/13. doi: 10.1002/14651858.CD009051.pub2 . 10.1002/14651858.CD009051.pub2 22786522 PMC6823267

[31] World Health Organization. Expanded programme on Immunization (‎EPI)‎ factsheet 2023: SEAR.2023. Available from: https://www.who.int/publications/i/item/SEAR-EPI-Factsheet-2023

[32] Metropolice the pathology specialist. HAV IgG antiody test. 2022. https://www.metropolisindia.com/parameter/hepatitis-a-virus-hav-hav-igg-antibody-serum

[33] Prinja S, Bahuguna P, Faujdar DS, Jyani G, Srinivasan R, Ghoshal S, et al. Cost‐effectiveness of human papillomavirus vaccination for adolescent girls in Punjab state: implications for India’s universal immunization program. Cancer. 2017;123(17):3253–3260. doi: 10.1002/cncr.30734 Epub 2017 May 4. . 10.1002/cncr.30734 28472550

[34] Goldie SJ, Gaffikin L, Goldhaber-Fiebert JD, Gordillo-Tobar A, Levin C, Mahé C, et al. Cost-effectiveness of cervical-cancer screening in five developing countries. New England Journal of Medicine. 2005;353(20):2158–2168. doi: 10.1056/NEJMsa044278 . 10.1056/NEJMsa044278 16291985

[35] Prinja S, Bahuguna P, Duseja A, Kaur M, Chawla YK. Cost of intensive care treatment for liver disorders at tertiary care level in India. PharmacoEconomics-open. 2018;2(2):179–190. doi: 10.1007/s41669-017-0041-4 10.1007/s41669-017-0041-4 29623618 PMC5972113

[36] Sudhindran S, Aboobacker S, Menon RN, Unnikrishnan G, Sudheer O, Dhar P. Cost and efficacy of immunosuppression using generic products following living donor liver transplantation in India. Indian Journal of Gastroenterology. 2012;31(1):20–23. doi: 10.1007/s12664-011-0138-0 . 10.1007/s12664-011-0138-0 22194185

[37] Chongmelaxme B, Phisalprapa P, Sawangjit R, Dilokthornsakul P, Chaiyakunapruk N. Weight reduction and pioglitazone are cost-effective for the treatment of non-alcoholic fatty liver disease in Thailand. Pharmacoeconomics. 2019;37:267–278. doi: 10.1007/s40273-018-0736-0 . 10.1007/s40273-018-0736-0 30430467

[38] Levy AR, Kowdley KV, Iloeje U, Tafesse E, Mukherjee J, Gish R, et al. The impact of chronic hepatitis B on quality of life: a multinational study of utilities from infected and uninfected persons. Value Health. 2008;11(3):527–538. doi: 10.1111/j.1524-4733.2007.00297.x Epub 2007 Dec 17.. 10.1111/j.1524-4733.2007.00297.x. 18179664

[39] Jyani G, Prinja S, Garg B, Kaur M, Grover S, Sharma A et al. Health-related quality of life among Indian population: The EQ-5D population norms for India. J of Glob Health.2023;13:04018. doi: 10.7189/jogh.13.04018 . https://doi.org/10.7189%2Fjogh.13.04018 36799239 PMC9936451

[40] Ministry of Statistics and Programme Implemetation. Consumer price index numbers on base 2012 = 100 for rural urban and combined for the month of November 2023. Available from: https://mospi.gov.in/sites/default/files/press_release/CPI_PR_12dec23.pdf

[41] Kwong S, Meyerson C, Zheng W, Kassardjian A, Stanzione N, Zhang K, et al. Acute hepatitis and acute liver failure: Pathologic diagnosis and differential diagnosis. Semin Diagn Pathol. 2019; 36(6):404–414. doi: 10.1053/j.semdp.2019.07.005 . 10.1053/j.semdp.2019.07.005 31405537

[42] Ramsay LC, Anyiwe K, Li M, Macdonald L, Coyte PC, Sander B. Economic evaluation of a publicly funded hepatitis A travel vaccination program in Ontario, Canada. Vaccine. 2019; 37(11):1467–1475. doi: 10.1016/j.vaccine.2019.01.070 . 10.1016/j.vaccine.2019.01.070 30770225

[43] International Monitary Fund. World economic outlook database. Available from: https://www.imf.org/en/Publications/WEO/weo-database/2022/October/download-entire-database

[44] Mauskopf JA, Sullivan SD, Annemans L, Caro J, Mullins CD, Nuijten M, et al. Principles of good practice for budget impact analysis: report of the ISPOR Task Force on good research practices—budget impact analysis. Value in health. 2007;10(5):336–347. doi: 10.1111/j.1524-4733.2007.00187.x 10.1111/j.1524-4733.2007.00187.x. 17888098

[45] Census of India 2011. Population projections for India and states 2011-2036.Report of the technical group on population projections, July 2020. 2020. Available from: https://main.mohfw.gov.in/sites/default/files/Population%20Projection%20Report%202011-2036%20-%20upload_compressed_0.pdf

[46] Teimouri F, Kebriaeezadeh A, Zahraei SM, Gheiratian M, Nikfar S. Budget impact analysis of vaccination against Haemophilus influenzae type b as a part of a Pentavalent vaccine in the childhood immunization schedule of Iran. Daru. 2017;25(1):1. doi: 10.1186/s40199-017-0166-0 10.1186/s40199-017-0166-0 28088246 PMC5237530

[47] World Health Organization. Monitoring vaccine wastage at country level-guidelines for programme manger. Available from: https://iris.who.int/handle/10665/68463

[48] Suwantika AA, Yegenoglu S, Riewpaiboon A, Tu HA, Postma MJ. Economic evaluations of hepatitis A vaccination in middle-income countries. Expert review of vaccines. 2013;12(12):1479–1494. Epub 2013/10/31. doi: 10.1586/14760584.2013.851008 . 10.1586/14760584.2013.851008 24168129

[49] Kar P. Is there a change in seroepidemiology of hepatitis A infection in India? The Indian Journal of Medical Research. 2006;123(6):727–729. Epub 2006/08/04. . 16885592

[50] Gurav Y, Babu GR, Vinu K, Lole K. Suspected spread of hepatitis A virus from a restaurant among adults in rural area of the Kerala state, India. Epidemiology & Infection. 2019; 147:e210. doi: 10.1017/S0950268819000967 10.1017/S0950268819000967. 31364560 PMC6624875

[51] Anonychuk AM, Tricco AC, Bauch CT, Pham B, Gilca V, Duval B, et al. Cost-effectiveness analyses of hepatitis A vaccine: a systematic review to explore the effect of methodological quality on the economic attractiveness of vaccination strategies. Pharmacoeconomics. 2008;26:17–32. doi: 10.2165/00019053-200826010-00003 10.2165/00019053-200826010-00003 18088156

[52] Luyten J, Van de Sande S, de Schrijver K, Van Damme P, Beutels P. Cost-effectiveness of hepatitis A vaccination for adults in Belgium. Vaccine. 2012;30(42):6070–80. Epub 2012/08/04. doi: 10.1016/j.vaccine.2012.07.049 . 10.1016/j.vaccine.2012.07.049 22858555

[53] PRS Lesislative Research. Kerala Budget Analysis 2023–2024. 2023. Available from: https://prsindia.org/files/budget/budget_state/kerala/2023/KL_State_Budget_Analysis_2023-24.pdf

[54] Ellis A, Rüttimann RW, Jacobs RJ, Meyerhoff AS, Innis BL. Cost-effectiveness of childhood hepatitis A vaccination in Argentina: A second dose is warranted. Revista Panamericana de Salud Publica/Pan American Journal of Public Health. 2007;21(6):345–56. doi: 10.1590/s1020-49892007000500002 . 10.1590/s1020-49892007000500002 17761046

[55] National Technical Advisory Group on Immunization (NTAGI). Minutes of the meetings of NTAGI, Ministry of Health and Family Welfare, Government of India. Available from: https://main.mohfw.gov.in/sites/default/files/MoM%20NTAGI%202016.pdf

