# Supplementary material for: Cost-effective analysis of hepatitis A vaccination in Kerala state, India
Source: PLoS One. 2024 Jun 27;19(6):e0306293. doi: 10.1371/journal.pone.0306293 (PMC11210869; doi:10.1371/journal.pone.0306293)
Supplement: S1 Text — (DOCX) [file pone.0306293.s001.docx]

**Supplemental file data**

**

**

**Supplemental Figure 1.** One-way sensitivity analysis results among individuals aged 15 years for live attenuated hepatitis A vaccination without screening strategy by A) societal perspective and B) payer perspective. For screening and live attenuated hepatitis A vaccination strategy by C) societal perspective and D) payer perspective.

**

**

**Supplemental Figure 2.** One-way sensitivity analysis results among individual aged 15 year for inactivated hepatitis A vaccination without screening strategy by A) societal perspective and B) payer perspective. For screening and inactivated hepatitis A vaccination strategy by C) societal perspective and D) payer perspective.

**

**

**Supplemental Figure 3.** One-way sensitivity analysis results among child aged 1 year for live attenuated hepatitis A vaccination by A) societal perspective and B) payer perspective. For inactivated hepatitis A vaccination by C) societal perspective and D) payer perspective.

**

**

**Supplemental Figure 4.** Cost-effectiveness plane by a payers perspective. A) among 15 years receiving inactivated hepatitis A vaccination without screening strategy B) among 15 years with screening and inactivated hepatitis A vaccination C) Child aged 1 year receiving inactivated hepatitis A vaccination

**

**

**Supplemental Figure 5.** Cost-effectiveness plane by a societal perspective A) among 15 years receiving live attenuated hepatitis A vaccination without screening strategy B) among 15 years with screening and live attenuated hepatitis A vaccination C) Child aged 1 year receiving live attenuated hepatitis A vaccination

**

**

**Supplemental Figure 6.** Cost-effectiveness plane by a payers perspective A) among 15 years receiving live attenuated hepatitis A vaccination without screening strategy B) among 15 years with screening and live attenuated hepatitis A vaccination C) Child aged 1 year receiving live attenuated hepatitis A vaccination

**

**

**Supplemental Figure 7.** Cost-effectiveness acceptability curves by a payers perspective. A) among 15 years receiving inactivated hepatitis A vaccination without screening strategy B) among 15 years with screening and inactivated hepatitis A vaccination C) Child aged 1 year receiving inactivated hepatitis A vaccination

**

**

**Supplemental Figure 8.** Cost-effectiveness acceptability curves by a societal perspective. A) among 15 years receiving live attenuated hepatitis A vaccination without screening strategy B) among 15 years with screening and live attenuated hepatitis A vaccination C) among 1 year receiving live attenuated hepatitis A vaccination

**

**

**Supplemental Figure 9.** Cost-effectiveness acceptability curves by a payers perspective. A) among 15 years receiving live attenuated hepatitis A vaccination without screening strategy B) among 15 years with screening and live attenuated hepatitis A vaccination C) among 1 year receiving live attenuated hepatitis A vaccination

**Supplemental Table**

**Supplemental Table 1. Cost-utility analysis results classified by live attenuated vaccine strategies and perspective among individuals aged 15 years**

| **Results** | **Societal perspective** | | | **Payer perspective results** | | |
| --- | --- | --- | --- | --- | --- | --- |
|  | **No vaccination** | **Live attenuated hepatitis A vaccination without screening** | **Screening and live attenuated hepatitis A vaccination** | **No vaccination** | **Live attenuated hepatitis A vaccination without screening** | **Screening and live attenuated hepatitis A vaccination** |
| Cost (₹) | 45,046.17 | 22,707.35 | 23,826.54 | 32,738.47 | 14,287.18 | 15,424.64 |
| LY^#^ | 24.17 | 24.51 | 24.30 | 24.17 | 24.51 | 24.30 |
| QALY^#^ | 18.64 | 18.93 | 19.44 | 18.64 | 18.93 | 19.44 |
| Incremental cost (₹) | NA | -22,338.82 | -21,219.64 | NA | -18,451.29 | -17,313.83 |
| Incremental LY^#^ | NA | 0.34 | 0.13 | NA | 0.34 | 0.13 |
| Incremental QALY^#^ | NA | 0.29 | 0.80 | NA | 0.29 | 0.80 |
| ICER^#^ (₹ per LY^#^ gained) | NA | -66,159 | -165,914 | NA | -54,646 | -135,375 |
| ICER^#^ (₹ per QALY^#^ gained) | NA | -76,964 | -26,563 | NA | -63,570 | -21,674 |

^#^Abbreviations: Life years (LY); Quality adjusted life years (QALY); Incremental cost-effectiveness ratio (ICER), Not applicable (NA)

**Supplemental Table 2 Cost-utility analysis results classified by live attenuated vaccine strategies and perspective among children aged 1 year**

| **Results** | **Societal perspective** | | **Payer perspective** | |
| --- | --- | --- | --- | --- |
|  | **No**  **vaccination** | **Live attenuated vaccine** | **No vaccination** | **Live attenuated vaccine** |
| Cost (₹) | 30,328.23 | 15,123.50 | 22,050.15 | 11,206.57 |
| LY^#^ | 27.16 | 27.18 | 27.16 | 27.17 |
| QALY^#^ | 21.68 | 21.84 | 21.68 | 21.84 |
| Incremental cost (₹) | NA | -15,204.72 | NA | -10,843.58 |
| Incremental LY^#^ | NA | 0.02 | NA | 0.02 |
| Incremental QALY^#^ | NA | 0.16 | NA | 0.16 |
| ICER^#^ (₹ per LY^#^ gained) | NA | -899,590 | NA | -607,399 |
| ICER^#^ (₹ per QALY^#^ gained) | NA | -95,448 | NA | -68,071 |

^#^Abbreviations: Life years (LY); Quality adjusted life years (QALY); Incremental cost-effectiveness ratio (ICER), Not applicable (NA)

**Supplemental Table 3.** Probabilistic sensitivity analysis results for individuals aged 15 year

| **Results** | **Societal perspective** | | | | | **Payer perspective results** | | | | |  |
| --- | --- | --- | --- | --- | --- | --- | --- | --- | --- | --- | --- |
|  | **No vaccination** | **Hepatitis A vaccination without screening** | | **Screening and hepatitis A vaccination** | | **No vaccination** | **Hepatitis A vaccination without screening** | | **Screening and hepatitis A vaccination** | | |
|  |  | **Live attenuated vaccine** | **Inactivated Vaccine** | **Live attenuated vaccine** | **Inactivated Vaccine** |  | **Live attenuated vaccine** | **Inactivated Vaccine** | **Live attenuated vaccine** | **Inactivated Vaccine** | |
| Cost (₹) | 44,412.98 | 22,311.48 | 27,618.34 | 23,452.43 | 27,516.89 | 32,901.39 | 14,034.18 | 20,573.37 | 15,246.15 | 20,254.25 | |
| LY^#^ | 24.19 | 24.51 | 24.58 | 24.31 | 24.35 | 24.20 | 24.53 | 24.59 | 24.32 | 24.36 | |
| QALY^#^ | 18.65 | 18.94 | 18.84 | 19.45 | 19.37 | 18.67 | 18.95 | 18.86 | 19.46 | 19.39 | |
| Incremental cost (₹) | NA | -22,101.51 | -16,794.64 | -20,960.56 | -16,896.09 | NA | -18,867.22 | -12,328.02 | -17,655.24 | -12,647.15 | |
| Incremental LY^#^ | NA | 0.36 | 0.39 | 0.12 | 0.17 | NA | 0.33 | 0.39 | 0.12 | 0.17 | |
| Incremental QALY^#^ | NA | 0.29 | 0.19 | 0.79 | 0.72 | NA | 0.29 | 0.19 | 0.79 | 0.72 | |
| ICER^#^  (₹ per LY^#^ gained) | NA | -67,020 | -42,953 | -173,564 | -100,625 | NA | -56,961 | -31,407 | -144,667 | -74,846 | |
| ICER^#^  (₹ per QALY^#^ gained) | NA | -77,503 | -88,390 | -26,422 | -23,453 | NA | -65,957 | -64,553 | -22,239 | -17,540 | |

^#^Abbreviations: Life years (LY); Quality adjusted life years (QALY); Incremental cost-effectiveness ratio (ICER), Not applicable (NA)

**Supplemental Table 4** Probabilistic sensitivity analysis results for individuals aged 1 year

| **Results** | **Societal perspective** | | | **Payer perspective** | | |  |
| --- | --- | --- | --- | --- | --- | --- | --- |
|  | **No vaccination** | **Live attenuated vaccine** | **Inactivated Vaccine** | **No vaccination** | **live attenuated vaccine** | **Inactivated Vaccine** | |
| Cost (₹) | 29,981.12 | 14,910.96 | 18,888.40 | 22,211.15 | 11,247.93 | 14,436.04 | |
| LY^#^ | 27.17 | 27.18 | 27.18 | 27.19 | 27.20 | 27.20 | |
| QALY^#^ | 21.69 | 21.84 | 21.84 | 21.71 | 21.87 | 21.85 | |
| Incremental cost (₹) | NA | -15,070.00 | -11,092.72 | NA | -10,963.22 | -7,775.11 | |
| Incremental LY^#^ | NA | 0.01 | 0.01 | NA | 0.01 | 0.01 | |
| Incremental QALY^#^ | NA | 0.16 | 0.16 | NA | 0.16 | 0.14 | |
| ICER^#^  (₹ per LY^#^ gained) | NA | -1,161,786 | -793,422 | NA | -789,730 | -522,490 | |
| ICER^#^  (₹ per QALY^#^ gained) | NA | -96,939 | -71,354 | NA | -70,196 | -55,296 | |

Abbreviations: Life years (LY); Quality adjusted life years (QALY); Incremental cost-effectiveness ratio (ICER)
